# Combination With Tomatidine Improves the Potency of Posaconazole Against *Trypanosoma cruzi*

**DOI:** 10.3389/fcimb.2021.617917

**Published:** 2021-03-04

**Authors:** Marianne Rocha-Hasler, Gabriel Melo de Oliveira, Aline Nefertiti da Gama, Ludmila Ferreira de Almeida Fiuza, Anna Frieda Fesser, Monica Cal, Romina Rocchetti, Raiza Brandão Peres, Xue Li Guan, Marcel Kaiser, Maria de Nazaré Correia Soeiro, Pascal Mäser

**Affiliations:** ^1^ Laboratório de Biologia Celular, Instituto Oswaldo Cruz (IOC/Fiocruz), Pavilhão Cardoso Fontes, Rio de Janeiro, Brazil; ^2^ Parasite Chemotherapy Unit, Swiss Tropical and Public Health Institute, Medical Parasitology and Infection Biology, Basel, Switzerland; ^3^ University of Basel, Basel, Switzerland; ^4^ Systems Biology of Lipid Metabolism in Human Health and Diseases Laboratory, Lee Kong Chian School of Medicine, Singapore, Singapore

**Keywords:** Chagas disease, tomatidine hydrochloride, drug combination, *T. cruzi*, lipid biosynthesis inhibitor

## Abstract

Azoles such as posaconazole (Posa) are highly potent against *Trypanosoma cruzi*. However, when tested in chronic Chagas disease patients, a high rate of relapse after Posa treatment was observed. It appears that inhibition of *T. cruzi* cytochrome CYP51, the target of azoles, does not deliver sterile cure in monotherapy. Looking for suitable combination partners of azoles, we have selected a set of inhibitors of sterol and sphingolipid biosynthetic enzymes. A small-scale phenotypic screening was conducted *in vitro* against the proliferative forms of *T. cruzi*, extracellular epimastigotes and intracellular amastigotes. Against the intracellular, clinically relevant forms, four out of 15 tested compounds presented higher or equal activity as benznidazole (Bz), with EC_50_ values ≤2.2 μM. Ro48-8071, an inhibitor of lanosterol synthase (ERG7), and the steroidal alkaloid tomatidine (TH), an inhibitor of C-24 sterol methyltransferase (ERG6), exhibited the highest potency and selectivity indices (SI = 12 and 115, respectively). Both were directed to combinatory assays using fixed-ratio protocols with Posa, Bz, and fexinidazole. The combination of TH with Posa displayed a synergistic profile against amastigotes, with a mean ΣFICI value of 0.2. *In vivo* assays using an acute mouse model of *T. cruzi* infection demonstrated lack of antiparasitic activity of TH alone in doses ranging from 0.5 to 5 mg/kg. As observed *in vitro*, the best combo proportion *in vivo* was the ratio 3 TH:1 Posa. The combination of Posa at 1.25 mpk plus TH at 3.75 mpk displayed suppression of peak parasitemia of 80% and a survival rate of 60% in the acute infection model, as compared to 20% survival for Posa at 1.25 mpk alone and 40% for Posa at 10 mpk alone. These initial results indicate a potential for the combination of posaconazole with tomatidine against *T. cruzi*.

## Introduction

Chagas disease (CD), a vector-borne anthropozoonosis endemic in the American continent, is caused by the protozoan parasite *Trypanosoma cruzi* (Chagas, 1909). The triatomine vector of CD is spread from the southern United States to the south of Argentina. Due to increasing global migration, CD has spread to other continents through a diversity of other transmission routes such as blood transfusion, organ transplantation, and mother-to-child (Gascon et al., 2010; Pérez-Molina and Molina, 2018). Also, oral transmission due to beverages contaminated with the feces or with infected triatomines currently represents a serious challenge in many endemic areas such as Brazil (Coura et al., 2014; Dias, 2017). This neglected disease presents a short acute phase with patent parasitemia, which is usually asymptomatic or oligosymptomatic with “flu-like” symptoms (Prata, 2001; Rassi et al., 2010). After six to nine weeks, parasite proliferation is controlled due to a competent immune response, and infected individuals enter a second stage, the chronic phase, with most of them remaining in an indeterminate form. However, after years or even decades, about 30% of the patients in the chronic phase develop progressive cardiac or gastrointestinal injuries (Ribeiro et al., 2012; Malik et al., 2015).

The front-line drugs for CD are two nitroderivatives, benznidazole (Bz) and nifurtimox. Both are far from ideal, with the occurrence of naturally resistant strains, lack of efficacy in the later chronic phase, and severe side effects that led to 10–30% therapy withdrawals (Molina et al., 2015). These limitations highlight an urgent need for novel, potent, and safer drugs for CD, and many strategies have been followed, including drug repurposing and drug combinations (Coura, 2009; Miranda and Sayé, 2019).

Drug combination may tackle more than one target simultaneously, allowing reduced doses, costs, time of drug administration, and reducing the risk of parasite drug resistance, providing increased efficacy and selectivity (Sun et al., 2016). These approaches have been largely explored in experimental models of Chagas disease (Batista et al., 2011; Diniz et al., 2013) as well as in clinical trials with chronic chagasic patients (Morillo et al., 2017).

Regarding drug repurposing, the identification of targets in *T. cruzi* shared by other pathogens fueled several *in vitro* and *in vivo* assays (Sales Junior et al., 2017). These piggy-back studies comprised fungal ergosterol biosynthesis inhibitors (EBIs) such as posaconazole (Posa) and E1224, the prodrug of ravuconazole (Sales Junior et al., 2017; Urbina, 2018), as well as a set of more specific inhibitors of the protozoan CYP51 orthologs, for instance VNI [(R)-N-(1-(2,4-dichlorophenyl)-2-(1H-imidazol-1-yl)ethyl)-4-(5-phenyl-1,3,4-oxadiazol-2-yl)benzamide)] and derivatives (Guedes-da-Silva et al., 2015). Unfortunately, both Posa and E1224 failed in clinical trials for CD, and many possibilities were raised regarding the lack of translation from the preclinical to clinical outcomes (Morillo et al., 2017; Chatelain and Ioset, 2018; Torrico et al., 2018).

A possible explanation for the disappointing outcome of the clinical trials with azole-type CYP51 inhibitors is, that these molecules fail to kill every single parasite, *i.e.*, they have high EC_99_ values. This notion is supported by *in vivo* (Francisco et al., 2015) and *in vitro* (Moraes et al., 2014; Cal et al., 2016; Fesser et al., 2020) models of pharmacodynamics. Nevertheless, azole-type CYP51 inhibitors have nanomolar EC_50_ values against *T. cruzi* and a high selectivity index, and they are well tolerated by the treated patients (Morillo et al., 2017). As a strategy to overcome the limitation of CYP51 inhibitors, we have proposed to combine them with a partner drug that either acts in the same pathway, sterol biosynthesis, or that inhibits a functionally linked pathway, sphingolipid synthesis (Fügi et al., 2015). Both rationales are supported by genetic interaction data from yeast (Eisenkolb et al., 2002; Guan et al., 2009; Fügi et al., 2015). Sphingolipids are a major class of lipids and ubiquitous constituents of eukaryotic membranes, playing also a role as bioactive signaling molecules involved in the regulation of cell growth, differentiation, senescence, and death (Pruett et al., 2008), as well as in virulence and survival of pathogens upon interaction with the host, including *T. cruzi* (Goldston et al., 2012; Guan and Mäser, 2017).

In the present work, we have assembled a panel of fifteen drugs and experimental compounds that interfere either with sterol synthesis or with sphingolipid metabolism. The compounds, their target enzymes, and their medical use (if any) are described in **Table 1**. All compounds were phenotypically assayed against the multiplicative forms of *T. cruzi*. Identified hit compounds were further combined with Posa and reference drugs in *in vitro* and *in vivo* models of parasite experimental infection.

**Table 1:** Compounds used in this study, their mode of action, and medical use.

	Chemical structure	Target/MoA	Indication/Use
**Reference drugs**			
Benznidazole [31593]	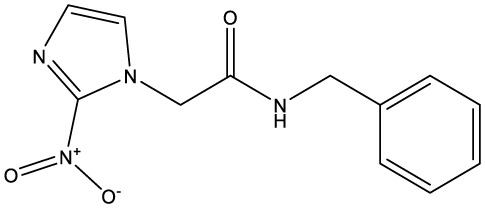	Oxydative stress after activation by nitroreductase I	Chagas disease
Fexinidazole [68792]	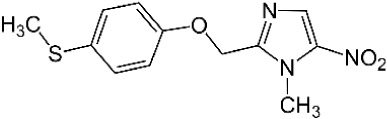	Oxydative stress after activation by nitroreductase I	Human African trypanosomiasis
**Lipid signaling**			
Bezafibrate [39042]	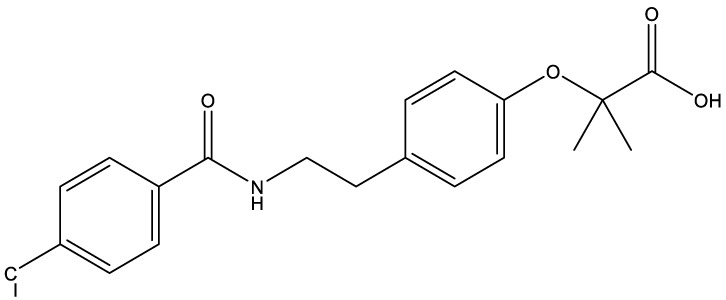	Peroxisome proliferator-activated receptor *α* agonist	Hyperlipidemia
D609 [4234241]	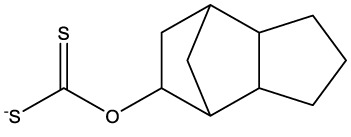	Phosphatidylcholine-specific phospholipase C	Experimental
FTY720 [107969]	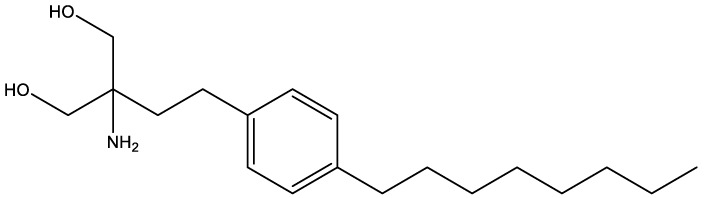	Parent molecule (Fingolimod) of FTY720-P	Multiple sklerosis, immuno-modulatory
FTY720-P [9908268]	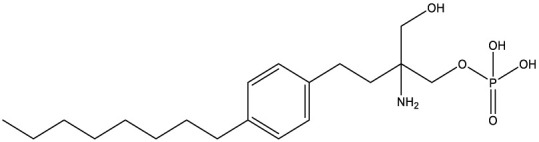	Sphingosine-1-phosphate-receptors	see above
**Sterol synthesis**			
Quinuclidine [7527]	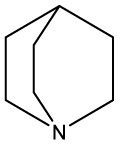	Derivatives inhibit squalene synthase (Erg9)	Experimental
3-Amino quinuclidine [123238]	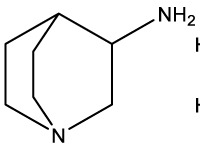	Derivatives inhibit squalene synthase (Erg9)	Experimental
Ro48-8071 [1949]	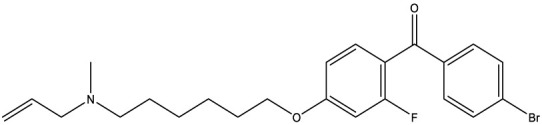	Oxidosqualene cyclase (Erg7)	Experimental
Posaconazole [468595]	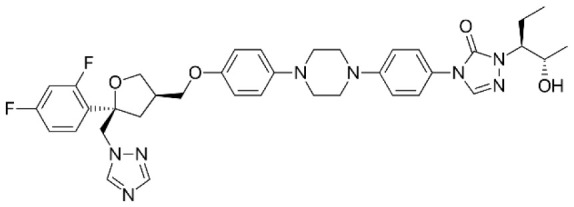	Lanosterol 14-*α* demethylase (Erg11)	Antifungal
Tomatidine [65576]	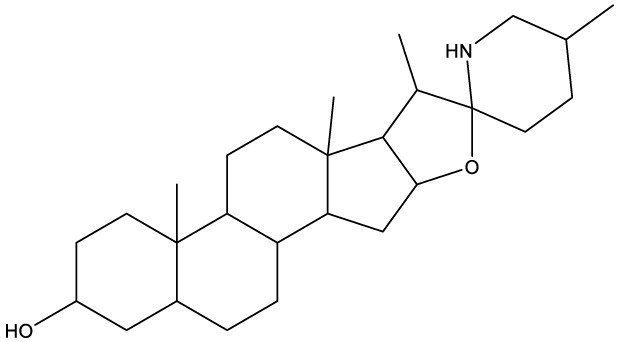	Sterol 24-C-methyltransferase (Erg6)	Natural dietary supplement, experimental
TMP-153 [125289]	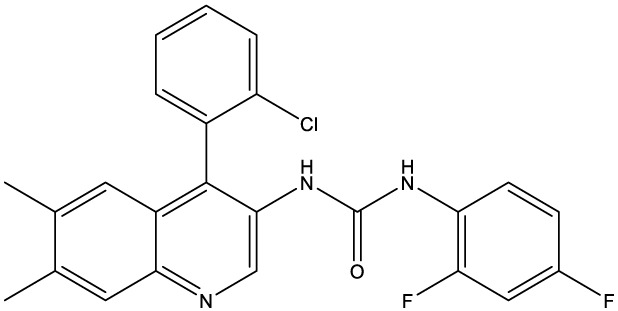	Sterol O-acyltransferase (ACAT)	Experimental
**Sphingolipid metabolism**			
Myriocin [6438394]	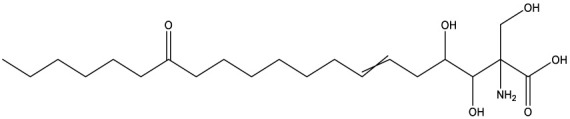	Serine palmitoyltransferase	Fungal toxin, experimental
Fumonisin B1 [2733487]	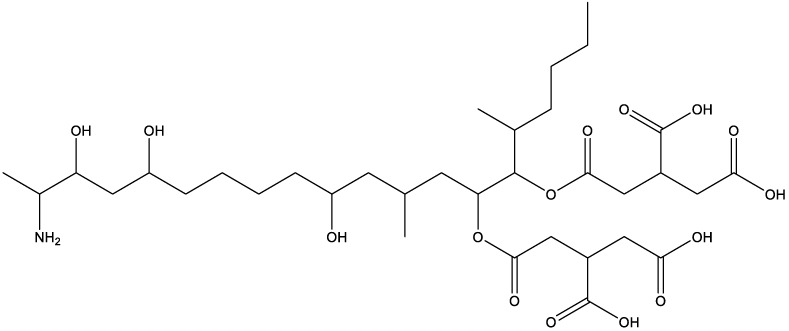	Ceramide synthase	Fungal toxin, experimental
PDMP [3129]	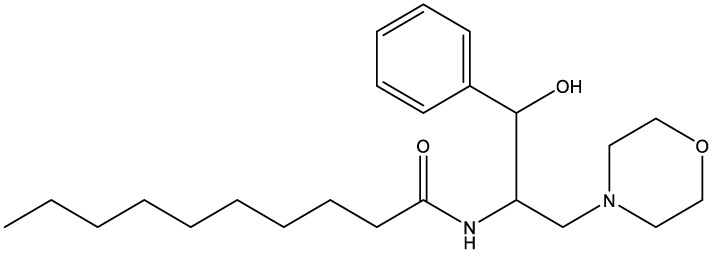	Glucosylceramide synthase	Experimental
AMP-Deoxynojirimycin [9822159]	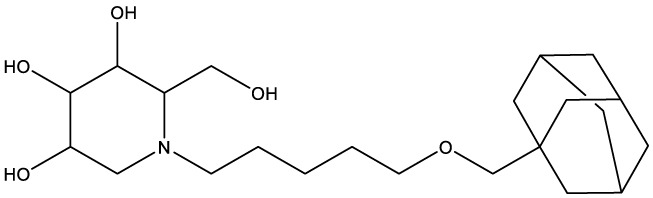	Non-lysosomal glucosylceramidase	Experimental
Amitriptyline [2160]	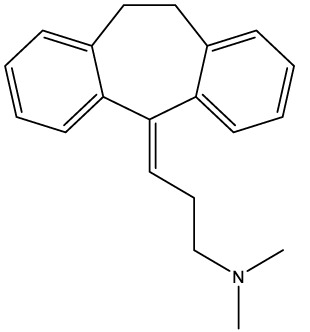	Acid sphingomyelinase	Depression
GW4869 [16078967]	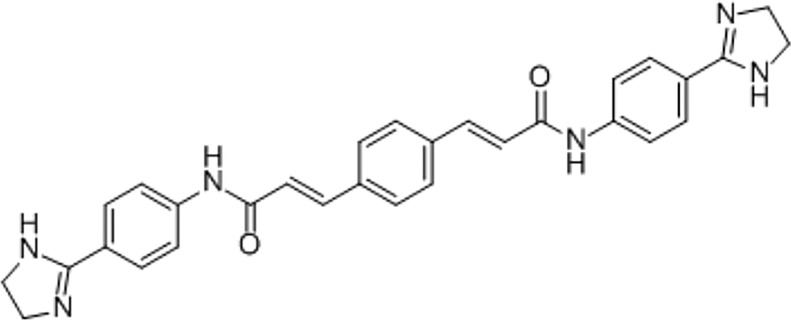	Neutral sphingomyelinase	Experimental

Numbers in brackets are PubChem compound ID numbers (pubchem.ncbi.nlm.nih.gov; Kim et al., 2021).

## Materials and Methods

### Compounds

Amitryptiline (hydrochloride), FTY720, FTY720 phosphate, AMP-deoxynojirimicin, D609 (potassium salt), fumonisin B1, myriocin, Ro48-8071, TMP-153, GW4869 (hydrochloride hydrate), PDMP (hydrochloride), tomatidine hydrochloride (TH) were purchased from Cayman chemicals and bezafibrate, quinuclidine hydrochloride, 3-aminoquinuclidine dihydrochloride, and posaconazole (Posa) were from Sigma-Aldrich Switzerland. Fexinidazole (Fexi) was received from DNDi and benznidazole (Bz) was purchased from Laboratório Farmacêutico do Estado de Pernambuco (Brazil). For *in vitro* tests, stock solutions of each compound were prepared in DMSO, never exceeding 0.6% DMSO as final concentration, which does not induce cellular damages to mammalian cells and parasites. For *in vivo*, Bz, Posa, and TH were prepared for oral (p.o., 100 µl) administration in extemporaneous solutions. For *in vivo* drug vehicles, Bz was diluted in distilled water with 3% Tween 80 (Sigma-Aldrich, Belgium), Posa and TH in aqueous solution of 0.5% carboxymethylcellulose (Sigma-Aldrich, Belgium).

### Mammalian Cells and Parasites for *In Vitro* Assays

L6 cells derived from rat skeletal myoblast (ATCC CRL-1458) were used as host cells for *T. cruzi* using trypomastigotes of Tulahuen C2C4 strain (DTU VI) expressing the *β*-galactosidase gene (LacZ) (Buckner et al., 1996). The cultures were sustained in RPMI-1640 supplemented with 10% inactivated fetal bovine serum (FBS) and 2 μM L-glutamine at 5% CO_2_ and 37°C. Epimastigote *T. cruzi* (STIB 980 strain, DTU TcI) were cultivated at 28°C in liver infusion tryptose (LIT) medium supplemented with hemin (2 μg/ml) and 10% FBS and were used in log phase of growth. Bloodstream trypomastigotes (Y strain, DTU II) were isolated from Swiss Webster mice infected at the peak of parasitemia (≥ 2.5 × 10^6^/ml), as reported (Meirelles et al., 1986).

### Activity on *T. cruzi* Epimastigotes

Epimastigotes (10^7^ parasites/ml) were incubated for 72 h at 28°C with serially diluted compound concentrations (eleven 1:3 dilutions) in supplemented LIT medium. Parasite viability and motility were evaluated by direct observation by light microscopy and fluorometric assays performed with resazurin (12.5 mg resazurin dissolved in 100 ml distilled water). After 2–4 h of incubation with resazurin solution, plates were read in Spectramax Gemini XS microplate fluorometer (Molecular Devices Cooperation, USA) using wavelengths of 536 nm (excitation) and 588 nm (emission) (Räz et al., 1997). Growth was expressed as percentage of the values of solvent-treated controls. The graphics program Softmax Pro (Molecular Devices) was used to construct dose–response curves and calculate EC_50_ (half maximal inhibitory concentration) values. Bz was used as reference drug.

### Activity on *T. cruzi* Intracellular Amastigotes

Rat skeletal myoblasts (L6 cell lines) were seeded in 96-well plates (10^4^ cells/well) in 100 μl RPMI 1640 medium supplemented with 10% fetal bovine serum (FBS) and 2 mM L-glutamine. The medium was removed after 24 h incubation and replaced by 100 μl of fresh medium containing 10^5^ LacZ trypomastigotes (Tulahuen DTU VI). After 48 h, the medium was again removed and replaced with or without a serial of compound concentrations (eleven three-fold dilution steps). After 96 h of compound exposure, CPRG/Nonidet (50 μl) substrate was added and the reading performed after 2–6 h at 540 nm in Versamax microplate reader (Molecular Devices Cooperation, USA). Growth was expressed as percentage of the values of solvent-treated controls. The graphics program Softmax Pro (Molecular Devices) was used to construct dose–response curves and calculate EC_50_ values. Bz was used as reference drug.

### Cytotoxicity Against L6 Cells

Rat skeletal myoblasts (L6 cell lines) were seeded in 96-well plates (4 × 10^4^ cells/well) in 100 μl RPMI 1640 medium supplemented with 10% fetal bovine serum (FBS) and 2 mM L-glutamine. After 24 h incubation the medium was replaced with 100 μl of fresh medium with or without a serial dilution of compound concentrations (eleven three-fold dilution steps). After 72 h of compound exposure, fluorescent dye resazurin (10 μl, 12.5 mg resazurin dissolved in 100 ml water) was added for 2 h and the readings performed at Spectramax Gemini XS microplate fluorometer (Molecular Devices Cooperation, USA), with excitation wavelength of 536 nm and an emission wavelength of 588 nm. Growth was expressed as percentage of the values of solvent-treated controls. The graphics program Softmax Pro (Molecular Devices) was used to construct dose–response curves and calculate EC_50_ values. Podophyllotoxin (a microtubule destabilizing agent) was used as positive control.

### Drug Combination

*In vitro* drug interactions on L6 cell cultures infected with Lac-Z *T. cruzi* using TH or Ro48-8071 combined to Bz, Posa or Fexi were performed at 1:3, 1:1, and 3:1 fixed-ratios (Fivelman et al., 2004) according to the same protocol as described above. Fractional inhibitory concentration indexes (FICI) and the sum of FICIs (∑FICI) were calculated as follows: FICI values = EC_50_ or EC_90_ of the combination/EC_50_ or EC_90_ of each compound alone. An overall ∑FICI was then determined and used to classify the nature of each interaction (Odds, 2003). ∑FICI ≤ 0.5 = synergism; 0.5 < ∑FICI ≤ 4.0 = additive (no interaction); ∑FICI > 4.0 = antagonism.

Isobolograms were built by plotting the FICI of compound 1 against the FICI of compound 2.

### Animals

Male Swiss Webster mice (18–23 g) were obtained from the Instituto de Ciência e Tecnologia em Biomodelos (ICTB/Fiocruz) (Rio de Janeiro, Rio de Janeiro, Brazil). Five mice were housed per cage, kept in a conventional room at 20–24°C under 12 h/12 h light/dark cycle. Sterilized water and chow were provided *ad libitum*. The animals were acclimated for 7 days before being used in the different assays. All procedures were done following Biosafety Guidelines in compliance with the Fiocruz and all animal procedure approved by the Committee of Ethics for the Use of Animals (CEUA L-38/17).

### Mouse Infection and Efficacy Studies

Male mice (n = 5 per group) were infected i.p. with 10^4^ bloodstream trypomastigotes of *T. cruzi* (Y strain). Only mice with positive parasitemia at day 5 post infection (dpi) were included in the studies. *T. cruzi*-infected mice were treated (p.o.) for ten consecutive days, from 5 to 14 dpi, with Posa (10 and 1.25 mg/kg body weight (mpk) corresponding to optimal and suboptimal doses of Posa for parasitemia suppression, respectively), TH (5–0.5 mpk) and combos of Posa plus TH, using the suboptimal dose of Posa (1.25 mpk) in different proportions, nearby those with best *in vitro* outcomes as follows: Posa 1.25 mpk + TH 5 mpk (ratio 1:4), Posa 1.25 mpk + TH 3.75 mpk (ratio 1:3) and Posa 1.25 mpk + TH 0.5 mpk (ratio 2.5:1)). Uninfected and *T. cruzi*-infected mice treated only with vehicle (aqueous solution of 0.5% carboxymethylcellulose) were age-matched and housed under identical conditions and used as controls (Simões-Silva et al., 2017). All compound formulations were freshly prepared before every administration.

### Parasitemia, Mortality Rates, and Endpoint

All animals were individually checked for circulating blood parasitemia by counting the number of parasites in 5 µl of blood taken from the tail vein and investigated under the microscope (Brener, 1962). Parasitemia was checked till 30 dpi, while mortality was checked daily up to 30 days after the administration of the last dose. Mortality was given as percentage of cumulative mortality (CM) (Simões-Silva et al., 2017).

### Statistical Analysis

All experiments were performed in triplicate in three independent experimental sets. The citotoxicity and antitrypanosomal activity were analyzed by ANOVA/Dunnet test using GraphPad Prism 5.01 software. *P* values of 0.05 or lower were assumed as significant.

## Results

The *in vitro* activity of the fifteen compounds (**Table 1**) was assessed on the multiplicative forms of *T. cruzi*: epimastigotes (strain STIB 980) and intracellular amastigotes (Tulahuen C2C4 strain expressing the *β*-galactosidase gene LacZ) (**Table 2**). In parallel, the cytotoxicity of the compounds was evaluated on mammalian host cells (**Table 2**). Against *T. cuzi* epimastigotes, two compounds (FTY720 and Ro48-8071) were promising, displaying similar potency as Bz (7.55, 11.6 and 13.9 µM, respectively; both of which were not significantly different to Bz, with *p values >*0.05 in comparison to Bz), and showing about 2.5–8-fold lower EC_90_ values than the reference drug (both with *p <* 0.05) (**Table 2**). Against the intracellular amastigotes, four compounds (FTY720, RO48-8071, tomatidine hydrochloride (TH) and TMP-153) displayed EC_50_ values ≤1 µM, lower than Bz (2.2 µM) (the four compounds presenting *p <*0.05 in comparison to Bz) (**Table 2**). Most of the tested compounds showed quite relevant toxicity towards mammalian host cells, leading to low selectivity indices (SIs), except for Ro48-8071 and TH, which presented promising SIs of 12 and 115, respectively (**Table 2**).

**Table 2:** Activity (EC_50_ and EC_90_, µM, n = 3) and selectivity of lipid biosynthesis inhibitors on *Trypanosoma cruzi* epimastigotes (STIB 980 strain) and intracellular amastigotes (Tulahuen strain in rat L6 myoblasts).

	Epimastigotes (mean ± SD) (µM)		Amastigotes (mean ± SD) (µM)		L6 cells (mean ± SD) (µM)	
Compound	EC_50_	EC_90_	EC_50_	EC_90_	EC_50_	SI^a^
Benznidazole	13.9 ± 2.2	101 ± 18	2.22 ± 0.98	6.61 ± 2.5	>384	>172
Bezafibrate	>276	>276	132.3 ± 38.6	226 ± 65	>276	>2.1
D609 (potassium salt)	154 ± 32	>375	99.4 ± 17.6	198 ± 32	247 ± 20	2.5
FTY720	7.55 ± 0.4	11.6 ± 0.3	0.81 ± 0.5	1.84 ± 1.3	0.39 ± 0.1	0.5
FTY720 Phosphate	>258	>258	93.8 ± 3.3	168 ± 25	127 ± 22	1.4
Quinuclidine hydrochloride	>677	> 677	478 ± 174	> 677	>677	>1.4
3-Amino quinuclidine dihydrochloride	>502	>502	268 ± 10	485 ± 28	>502	>1.8
Ro48-8071	11.6 ± 6.1	29.4 ± 16.2	0.47 ± 0.09	2.5 ± 1.9	5.7 ± 2.4	12
Posaconazole	>14.2	>14.2	0.002 ± 0.001	0.016 ± 0.005	>1.42	>700
Tomatidine (hydrochloride)	192 ± 8.1	>221	0.78 ± 0.2	1.8 ± 0.2	89.5 ± 26.7	115
TMP-153	106 ± 27	>228	0.09± 0.04	3.9 ± 2.1	0.12 ± 0.03	1.4
Myriocin	>249	>249	130 ± 34	231 ± 25	>249	>1.9
Fumonisin B1	>92	>92	50.8 ± 5.7	84 ± 9.1	>92	>1.8
PDMP (hydrochloride)	47.9 ± 9.4	88.7 ± 18	12.5 ± 2.9	24.6 ± 9	33.5 ± 3.1	2.7
AMP-Deoxy nojirimycin	158 ± 5.9	237 ± 2	34.8 ± 15.1	66.6 ± 15.4	40.4 ± 10.9	1.2
Amitriptyline (hydrochloride)	59.9 ± 5.4	96.8 ± 3.3	12.2 ± 3.6	20.9 ± 3	17.2 ± 1.2	1.4
GW4869 (hydrochloride hydrate)	>173	>173	106 ± 44	>173	>173	>1.7

^a^Selectivity Index based on the EC_50_ and EC_50_ on intracellular amastigotes and the mammalian cells, respectively.

**Figure 1:**
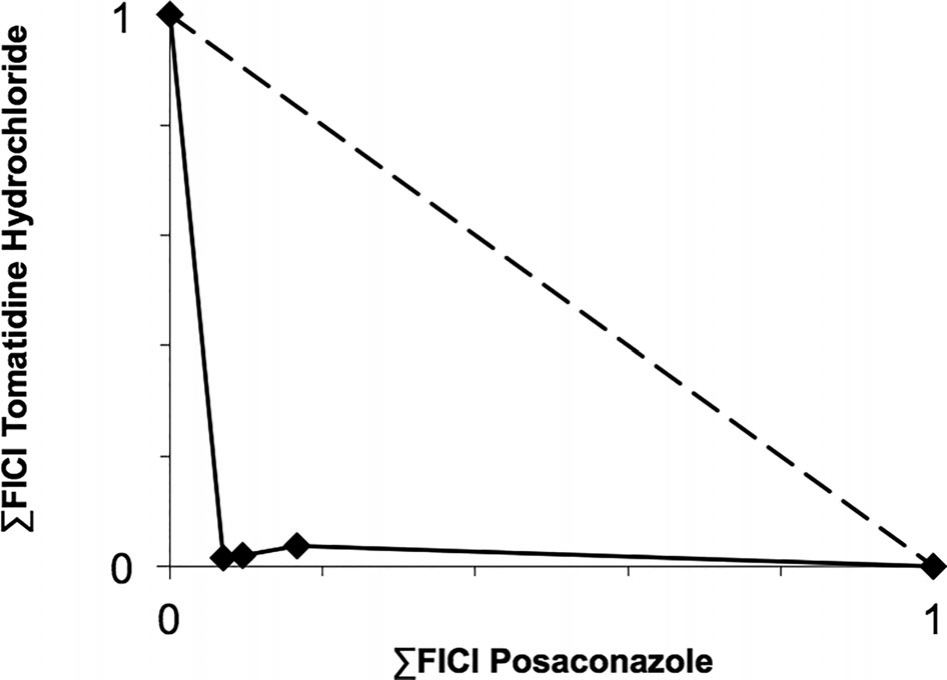
Isobologram showing the sum of the fractional inhibitory concentration indexes (∑FICI) of tomatidine hydrochloride and posaconazole, demonstrating the synergistic interaction of the combination in different fixed ratio proportions (1:3, 1:1, and 3:1) against *T. cruzi* intracellular amastigotes (strain Tulahuen in L6 cells). ∑FICI ≤ 0.5 = synergism; 0.5 < ∑FIC ≤ 4.0 = additive (no interaction); ∑FICI > 4.0 = antagonism.

Based on their high activity against intracellular amastigote *T. cruzi* and good selectivity towards the mammalian host cells, Ro48-8071 and tomatidine were moved to *in vitro* combination assays with the reference drug for CD (Bz) and two others that displayed efficacy in *in vitro* and *in vivo* assays of *T. cruzi* experimental infection: the imidazole CYP51 inhibitor Posa and the nitroimidazole Fexinidazole (Fexi) (**Table 3**). Of the six combinations tested, only that between TH and Posa had a synergistic profile with mean ΣFICI values below 0.5, based on their EC_50_ (**Table 3**, **Figure 1**). These results encouraged us to follow up with *in vivo* studies. Posa or TH did not show any signs of toxicity when administered to female mice p.o. up to 200 mpk (data not shown).

**Table 3:** Sum of the mean fractional inhibitory concentration indices (ΣFICI) of the combinations between Ro48-8071 or tomatidine hydrochloride and benznidazole, posaconazole, or fexinidazole in different fixed ratio proportions (1:3, 1:1, and 3:1; the first number corresponds to the standard drug) against *T. cruzi* intracellular amastigotes (strain Tulahuen in L6 cell lines).

Combos Mean ΣFICIs	Benznidazole				Posaconazole				Fexinidazole			
	Ro48-8071		Tomatidine hydrochloride		Ro48-8071		Tomatidine hydrochloride		Ro48-8071		Tomatidine hydrochloride	
	**EC_50_**	**EC_90_**	**EC_50_**	**EC_90_**	**EC_50_**	**EC_90_**	**EC_50_**	**EC_90_**	**EC_50_**	**EC_90_**	**EC_50_**	**EC_90_**
1:3	1.8	1.5	0.7	1.0	1.1	0.8	0.1	0.4	1.4	1.2	1.4	0.9
1:1	4.0	3.3	1.1	1.1	1.6	1.7	0.2	0.7	3.6	3.1	1.1	0.9
3:1	4.0	5.0	1.7	1.3	4.4	3.5	0.3	0.4	5.5	5.0	1.7	1.7

ΣFICI ≤ 0.5 synergism; 0.5 < ΣFICI ≤ 4 additive (no interaction); ΣFICI > 4 antagonism.

**Figure 2:**
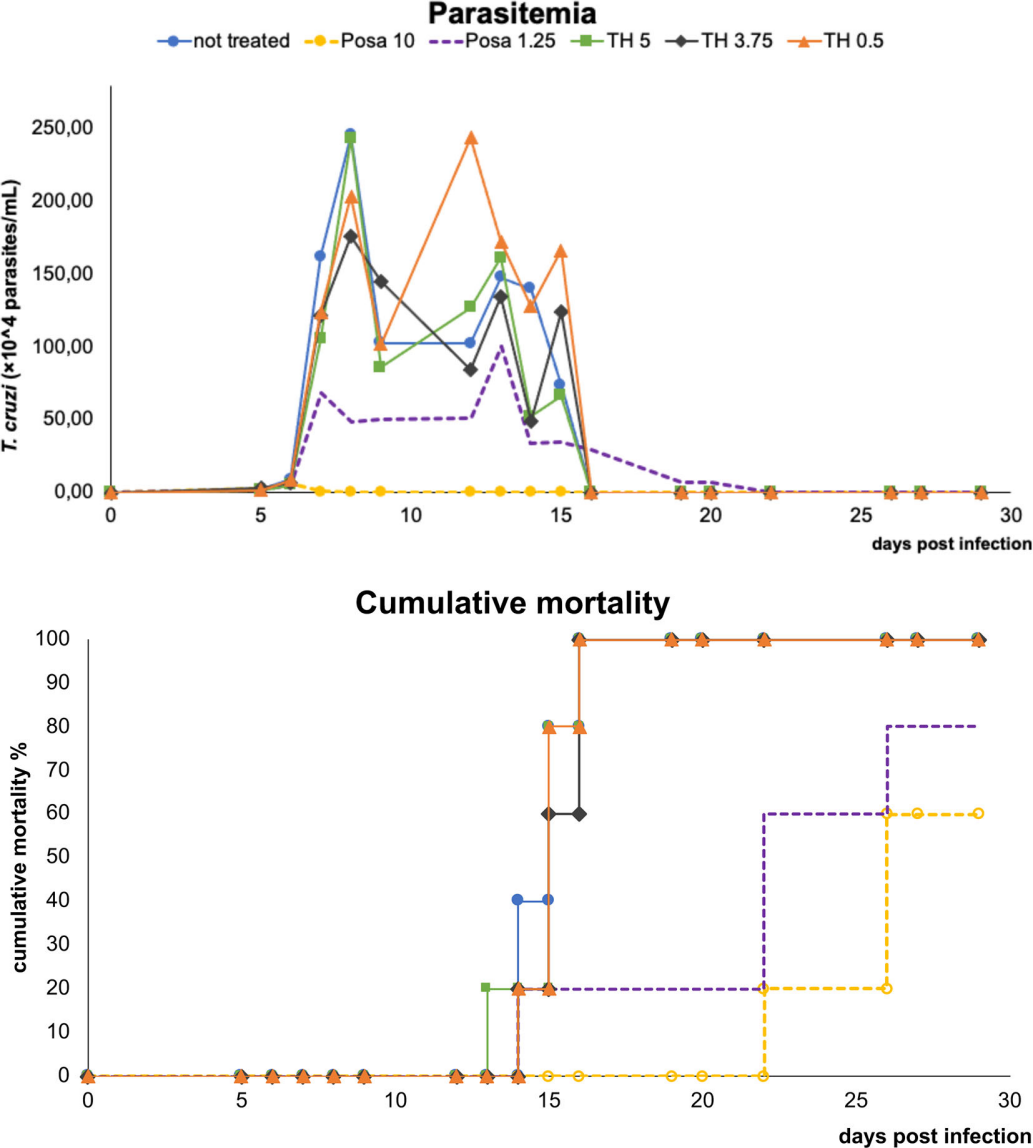
Parasitemia and cumulative mortality of mice infected with *T. cruzi* (Y strain) treated with vehicle alone, posaconazole (Posa 10 and 1.25 mpk), or tomatidine hydrochloride (TH 5, 3.75 and 0.5 mpk) administrated for ten consecutive days (dpi 5 to 14).

Before moving to co-administration schemes, TH and Posa alone were administered in mouse models of acute *T. cruzi* infection (**Figure 2**). Posa at 10 mpk suppressed parasitemia on the peak (8 dpi), providing 40% survival of mice, while all vehicle-treated mice died until the endpoint (**Figure 2**). A suboptimal dose of Posa (1.25 mpk) decreased the parasitemia peak (about 80%), but only provided a mild protection against mortality (20% of animal survival) (**Figure 2**). On the other hand, all tested doses of TH (up to 5 mpk) alone resulted in a maximum reduction of only 28% in blood parasitemia on the peak and were unable to protect against the mortality induced by the infection since all animals died by 20 dpi (**Figure 2**).

**Figure 3:**
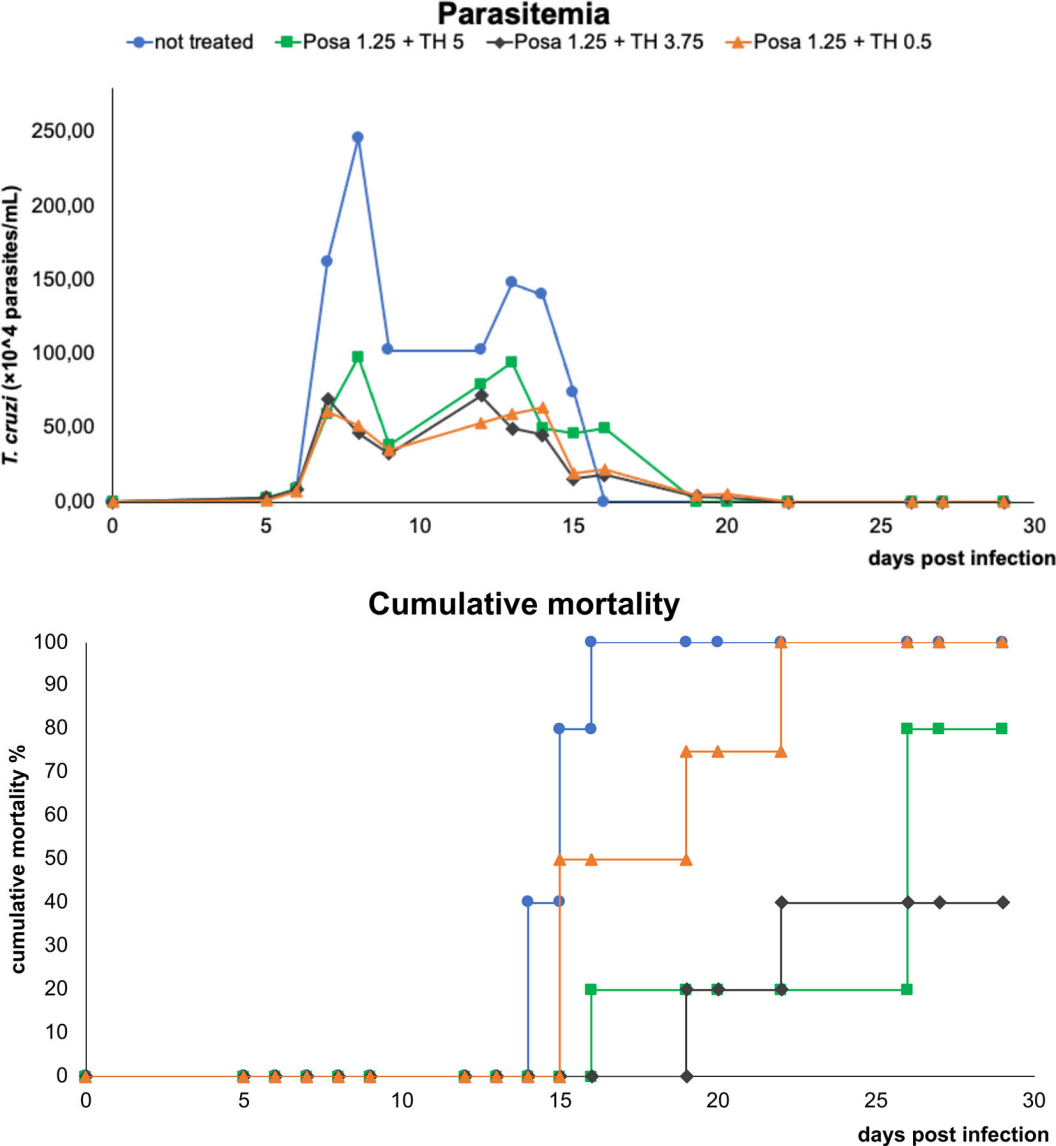
Parasitemia and cumulative mortality of mice infected with *T. cruzi* (Y strain) treated with vehicle or different co-administration proportions of posaconazole and tomatidine hydrochloride (Posa 1.25 + TH 5, Posa 1.25 + TH 3.75 and Posa 1.25 + TH 0.5 mpk) for ten consecutive days (dpi 5 to 14).

The co-administration of TH (variable doses from 0.5 to 5 mpk) plus Posa at the suboptimal dose of 1.25 mpk led to a parasitemia drop ranging from 60 to 80%, and cumulative death ranging from 40 to 100% (**Figure 3**). The best effect as evaluated by the concomitant reduction in peak parasitemia (80%) and increased animal survival (60%) was achieved with the combo Posa 1.25 mpk + TH 3.75 mpk (ratio 1:3) (**Figure 3**), which corroborated the best *in vitro* ratio of combination (**Table 3**).

## Discussion

Drug repurposing and drug combination are pre-clinical strategies used in experimental pharmacology to tackle many diseases, reaching quite effective results when applied to clinical trials and off-label use (Sbaraglini et al., 2016). Successful examples from neglected tropical diseases include the repositioning of nifurtimox administered in combination with eflornithine for Human African trypanosomiasis (Priotto et al., 2007), the association of miltefosine and paromomycin, and sodium stibogluconate plus paromomycin, for visceral leishmaniasis (Atia et al., 2015; Rahman et al., 2017; Alves et al., 2018). Regarding Chagas disease, fungicidal inhibitors of CYP51 enzymes have been assayed in clinical trials (e.g., Posa and E1224 in association with Bz), but unfortunately had high rates of therapeutic failure (Morillo et al., 2017; Torrico et al., 2018). On the other hand, the BENEFIT trial demonstrated that, although effective to reduce parasite load in chronic chagasic patients, Bz did not impair the progression of cardiac damages, reinforcing the need to search for new therapeutic alternatives for CD (Rassi et al., 2017).

Inhibitors of sterol biosynthetic enzymes and sphingolipid metabolism and signaling had been proposed as combination partners for Posa (Fügi et al., 2015). Selected inhibitors (**Table 1**) were phenotypically assessed against *T. cruzi*. FTY720 and Ro48-8071 were as active as Bz against epimastigotes. Against the therapeutically relevant intracellular forms, four compounds had similar or even higher potency than Bz: FTY720, Ro48-8071, tomatidine hydrochloride and TMP-153. The different EC_50_ values of the studied compounds (including the reference drug) against epimastigotes and amastigotes can be explained by the distinct cellular metabolism of these proliferative forms that face different environments and hosts. Although we cannot exclude variabilities in drug susceptibility among the different strains (Zingales et al., 2014; Zingales, 2018), our data confirm the importance of using the intracellular amastigote form for drug discovery (Romanha et al., 2010; de Castro et al., 2011).

Interestingly, tomatidine hydrochloride (TH) had shown activity against bacteria (*Staphylococcus aureus*), fungi (*Candida albicans*), Chikungunya, Dengue, and Zika virus, and the trypanosomatids *Phytomonas serpens* and *Leishmania amazonensis* (Mitchell et al., 2011; Mitchell et al., 2012; Robbins et al., 2015; Dorsaz et al., 2017; Soltani et al., 2017; Diosa-Toro et al., 2019; Troost et al., 2020). While ATP synthase was proposed as the target of TH in *S. aureus* (Lamontagne Boulet et al., 2018), the antifungal and antitrypanosomal target of TH turned out to be C-24 sterol methyltransferase (Medina et al., 2012; Medina et al., 2015; Dorsaz et al., 2017). This enzyme, encoded by the gene ERG6, catalyzes a downstream step of ergosterol synthesis from CYP51 (ERG11). Ro48-8071 targets lanosterol synthase (ERG7), the step immediately before CYP51. In a previous study (PubMed id 9491766, Chataing et al., 1998), tomatidine at 5.7 µM inhibited the growth of *T. cruzi* epimastigote cultures to 50% after four days of incubation.

Based on their promising activity and selectivity against *T. cruzi* amastigotes, Ro48-8071 and TH were selected for *in vitro* combination testing using fixed-ratio proportions as reported (Simões-Silva et al., 2016). Regarding the choice of partner drugs, Bz as one of the standard drugs for CD was an obvious candidate (Coura, 2009). Posa and Fexi are very potent anti-*T. cruzi* agents *in vitro* and *in vivo* (Urbina, 2015) that were moved to clinical trials for CD (Bahia et al., 2012; Morillo et al., 2017; DNDi Portfolio, 2020). None of the combos made with Ro48-8071 showed synergistic activity and were therefore not further investigated. Combos of Bz and Fexi with TH were also additive. However, the association of TH plus Posa was essentially synergistic, displaying ∑FICI = 0.2.

A synergistic interaction for TH has already been reported with aminoglycoside antibiotics, being more effective in inhibiting colony growth of *S. aureus* clinical isolates as compared to standard monotherapies (Mitchell et al., 2011; Mitchell et al., 2012; Soltani et al., 2017). Also, the combination of TH with fluconazole exhibited synergistic interaction against a *C. albicans* azole-resistant strain (Robbins et al., 2015), thus confirming the potential of TH for drug combination protocols.

Based on these *in vitro* results, Posa + TH was moved to *in vivo* assays using a well-established mouse model of acute *T. cruzi* infection (Romanha et al., 2010). TH alone did not present antiparasitic activity *in vivo*. However, it is important to note that the lack of *in vivo* efficacy may be due to the poor solubility of TH. Previous studies reported that, as TH is a highly hydrophobic sterol-like molecule, many vehicles including DMSO, ethanol or cyclodextrin failed to demonstrate efficacy in *in vivo* models of *C. albicans* infection, except for the use of a nanoparticle-based formulation that allowed successful reduction of fungal burden in infected mice (Dorsaz et al., 2017). Thus, the exploration of other formulations for TH is desirable to better assess its potential against *T. cruzi in vivo*. When the combos were assayed, the best results in terms of reduction of parasitemia and mortality were obtained with the proportion of Posa 1.25 mpk + TH 3.75 mpk, which correlates to the most synergistic combo *in vitro* (drug ratio 1:3). The combination of Posa at 1.25 mpk plus TH at 3.75 mpk displayed a survival rate of 60% in the acute infection model as compared to 20% for Posa at 1.25 mpk alone, and 40% for Posa at 10 mpk alone.

Thus, our finding that only the combo of Posa plus TH gave a synergistic profile *in vitro* was further corroborated by our *in vivo* assays demonstrating a reduction in parasite load and animal death rates. These results possibly are due to the simultaneous action on enzymes (lanosterol 14-*α* demethylase and sterol 24-C-methyltransferase) to the sterol biosynthetic route, impacting the fitness profile and metabolism of the intracellular, clinically relevant form of *T. cruzi*.

TH is a natural compound, originally found in unripe tomatoes, that has a wide array of bioactivities including antioxidant, anticarcinogenic and antimicrobial effects (Friedman, 2013). TH exerts antifungal and antitrypanosomatid effects by inhibition of C-24 sterol methyltransferase (Medina et al., 2015; Dorsaz et al., 2017). The finding that TH synergistically interacts with Posa encourages further studies with this class of compound and reinforces the potential of drug repurposing and combination protocols. These approaches represent reduced cost and time in the search for better treatments for CD, which is clearly relevant considering the shortage of resources in benefit of the poor population around the world affected by neglected tropical diseases such as Chagas disease. Although Posa at 10 mpk and the combo Posa 1.25 mpk + TH 3.75 mpk suppressed/highly reduced the parasitemia, neither therapeutic scheme was able to reach 100% animal survival and induce sterile cure. Thus, further studies will need to address the efficacy against dormant forms of *T. cruzi* as recent data suggest the existence of an adaptive difference between parasite strains to generate dormant cells, and that homologous recombination in *T. cruzi* may be important for dormancy stages (Sánchez-Valdéz and Padilla, 2019; Resende et al., 2020).

## Data Availability Statement

The raw data supporting the conclusions of this article will be made available by the authors, without undue reservation.

## Ethics Statement

All procedures followed the guidelines in compliance with the Fiocruz Committee of Ethics for the Use of Animals (CEUA L-38/17).

## Author Contributions

MR-H performed the *in vitro* and *in vivo* studies, data analysis, and drafted the manuscript. PM, MK, and MS (corresponding author) obtained the funding, conceived the study, performed data analysis, and drafted the manuscript. XG helped in drafting the manuscript and in study design. GO, AD, LF, and RP contributed to the *in vivo* studies. AF, MC, and RR contributed to the *in vitro* studies. All authors contributed to the article and approved the submitted version.

## Funding

The fundings were provided by the Swiss National Science Foundation (SNF grant IZRJZ3_164172), Fundação Carlos Chagas Filho de Amparo à Pesquisa do Rio de Janeiro (FAPERJ), Coordenação de Aperfeiçoamento de Pessoal de Nível Superior (CAPES), Conselho Nacional de Desenvolvimento Científico e Tecnológico (CNPQ), PAEF, and Instituto Oswaldo Cruz (IOC/Fiocruz). MS is CNPq fellow and Cientista do Nosso Estado CNE FAPERJ.

## Conflict of Interest

The authors declare that the research was conducted in the absence of any commercial or financial relationships that could be construed as a potential conflict of interest.
